# Colistin resistant *Escherichia coli* carrying *mcr*-*1* in urban sludge samples: Dhaka, Bangladesh

**DOI:** 10.1186/s13099-017-0227-4

**Published:** 2017-12-20

**Authors:** Aminul Islam, Zillur Rahman, Shirajum Monira, Md. Anisur Rahman, Andrew Camilli, Christine M. George, Niyaz Ahmed, Munirul Alam

**Affiliations:** 1 International Center for Diarrhoeal Disease Research, Bangladesh (icddr,b), Mohakhali, Dhaka, 1212 Bangladesh; 2 Department of Biotechnology and Genetic Engineering, Noakhali Science and Technology University, Noakhali, 3814 Bangladesh; 3 Department of Molecular Biology and Microbiology, Howard Hughes Medical Institute, TuftsUniversity School of Medicine, Boston, USA; 4 Johns Hopkins Bloomberg School of Public Health, Baltimore, USA

**Keywords:** Colistin resistant, *Escherichia coli*, *mcr*-*1*, Bangladesh, First report

## Abstract

Of 48 bacteria belonging to the family *Enterobacteriaceae* tested from urban sludge samples, one *Escherichia coli* isolate was resistant to colistin and possessed the resistance marker gene *mcr*-*1* found for the first time from Bangladesh. The colistin resistant *E. coli* was multidrug resistant showing resistance to 11 different antibiotics tested.

## Background

Antimicrobial resistance is a multi-sectoral problem which is now recognized as one of the most serious threats to human health globally. Resistance trends among *Enterobacteriaceae* are especially worrisome considering their ubiquity in the environment and animal systems, and their enhanced propensity to acquire antibiotic resistance determinants through mobile genetic elements. Indiscriminate use of antibiotics is mainly responsible for the emergence of *Enterobacteriaceae* resistant to multiple antibiotics including carbapenems.

Colistin, a cationic polypeptide, is considered as one of the last-resort drugs of choice for the treatment of multi-drug resistant, Gram negative bacteria such as carbapenem resistant *Enterobacteriaceae* (CRE). Present day increase in the incidences of multi-drug resistant bacteria has resulted in enhanced use of colistin globally, with an inevitable risk of emerging resistance. A study in Vietnam has shown increasing resistance in commensal *Escherichia coli* associated with the extensive use of colistin in livestock and poultry industry [1]. A recent study has shown colistin sulfate to be the most commonly used antibiotic in poultry industry in Bangladesh [2]. Acquired resistance to colistin is generally associated with chromosomal mutations [3], although a new plasmid-mediated transferable resistance determinant, the *mcr*-*1* gene, encoding a phosphoethanolamine transferase, has been described recently in China [4]. Since the plasmid encoding *mcr*-*1* has been established as a marker for colistin resistance, *Enterobacteriaceae* carrying this gene has been reported from many parts of the world [5]. Considering the widespread occurrence of colistin resistance and the impending danger associated with it, we screened bacteria belonging to the family *Enterobacteriaceae* isolated from sludge samples of Dhaka city for their resistance to colistin and for the presence of colistin resistance-related gene *mcr*-*1*. Here we report the occurrence of colistin resistant *E. coli* carrying *mcr*-*1* gene in urban environment of Dhaka, Bangladesh.

## Methods

After preliminary identification following standard culture methods and final biochemical confirmation with API 20 E (bioMérieux, France), 48 bacterial isolates including *Escherichia coli* (n = 23), *Klebsiella pneumoniae* (n = 15), *Pseudomonas luteola* (n = 6), *Pseudomonas aeruginosa* (n = 1), *Pantoea* spp. (n = 2) and *Citrobacter freundii* (n = 1), collected from sludge samples of Dhaka city, were tested for their response to colistin by measuring the minimum inhibitory concentration (MIC) by E-test (Biomeuriex). The results were interpreted according to European Committee on Antimicrobial Susceptibility Testing (EUCAST) breakpoints [6]. Although broth microdilution assay is recommended by EUCAST for determining MIC, several studies have found an unequivocal correlation between the Etest and reference techniques [7–9].

Bacterial DNA was obtained from all isolates by the standard boiling method. Detection of *mcr*-*1* gene was performed by polymerase chain reaction (PCR) using the primer as described elsewhere [4]. Amplified fragment of *mcr*-*1* was sequenced using an ABI PRISM BigDye Terminator Cycle Sequencing Reaction kit (Applied Biosystems) on an ABI PRISM 310 automated sequencer (Applied Biosystems). After analyzing the raw sequence with sequence analysis software (Chromas), a deduced 252 bp sequence was then searched for homology by Basic Local Alignment Search Tool (BLAST). The partial sequence of the gene was submitted to GenBank (Accession Number MF173059).

## Results and discussion

Among all the tested bacteria, one of the *E. coli* isolates was found resistant to colistin with a determined MIC value of 4 μg/mL. This colistin resistant *E. coli* isolate called E3B was then tested for its response to 15 different antibiotics by Kirby–Bauer disk diffusion method [10], following the Clinical Laboratory Standards Institute recommendations. Our results confirmed the colistin resistant *E. coli* to be multidrug resistant (MDR) as the isolate E3B was resistant to 10 different antibiotics: Nalidixic acid, Ciprofloxacin, Levofloxacin, Azithromycin, Gentamicin, Erythromycin, Sulfamethoxazole/Trimethoprim, Ampicillin, Cephalothin and Tetracycline, but sensitive to Mecillinam, Fosfomycin, Ceftriaxone, Cefixime and Meropenem. This appears to be an alarming trend for a populous country like Bangladesh where infection control is becoming increasingly challenging due to the rise of multidrug resistance among members of the family *Enterobacteriaceae*, including *E. coli* [11, 12].

All of the 48 bacterial isolates, including the colistin resistant *E. coli* were screened by PCR for the presence of colistin resistance gene *mcr*-*1* using specific primers. However, only the colistin resistant *E. coli* isolate E3B supported the amplification of a 309 bp fragment of *mcr*-*1*. The PCR amplicon of the *mcr*-1 gene was then sequenced. Although it was not possible for us to cover the full length sequence of the gene, BLAST homology searching demonstrated the deduced sequenced data of the 252 bp DNA fragment (Accession No. MF173059) to be identical to that of the *mcr*-*1* reported from *E. coli* strain SHP45 (GenBank Accession No. KP347127) isolated from a Chinese pig farm [4]. To our knowledge, colistin resistant *E. coli* carrying *mcr*-*1* has not been isolated so far from hospital settings, and this is the first report of the occurrence of colistin resistant *E. coli* carrying resistant marker *mcr*-*1* from environment of Bangladesh.

Finally, the data presented in this study show environmental dissemination of MDR *E. coli* carrying colistin resistance and related marker gene *mcr*-*1* via urban sludge disposed into the water bodies. Dhaka is a densely populated city with circular river systems, and millions living in urban slums do not have access to safe drinking water. Given this, fecal–oral transmission might allow MDR enteric pathogens to transmit rapidly. Further, the high selection pressure of residual antibiotics in the urban environment, and since the colistin resistance marker *mcr*-*1* can be transferred horizontally; there is an urgent requirement for broader surveillance in both clinical and environmental settings in Bangladesh in order to prevent further spreading of this resistance gene.
